# Advanced nano modification of ecofriendly glauconite clay for high efficiency methylene blue dye adsorption

**DOI:** 10.1038/s41598-024-71979-y

**Published:** 2024-10-09

**Authors:** Eman M. Saad, Manar Wagdy, Adel S. Orabi

**Affiliations:** 1 Chemistry Department, Faculty of Science, Suez University, Suez, Egypt; 2 Chemistry Department, Faculty of Science, Suez Canal University, Ismailia, Egypt

**Keywords:** Dye removal, BMNC, Adsorption isotherm, Kinetic, Thermodynamics, In silico, Environmental sciences, Chemistry, Materials science

## Abstract

This research focuses on the utilization of nano glauconite clay as an environmentally friendly sorbent for the removal of cationic dyes, particularly Methylene Blue (MB), from polluted water. The glauconite clay was sourced from the El Gidida region of Egypt and subjected to grinding in a laboratory-type ball mill to ensure homogeneity and increase the active sites available for the adsorption process. The resulting ball milled nano clay (BMNC) was characterized using techniques such as X-ray fluorescence (XRF), Fourier transform infrared (FT-IR) spectroscopy, transmission electron microscopy (TEM), energy dispersive X-ray spectroscopy (EDS), and X-ray diffraction (XRD). The concentration of MB dye was monitored using UV–Vis spectroscopy to assess the adsorption capacity of BMNC under various conditions including pH, time, dose, and temperature. The optimal conditions for the adsorption process were determined to be a pH range of 7–8, a contact time of 60 min, and a dose of 200 ppm, resulting in an adsorption capacity of 128 mg/g. This process demonstrated both low cost and high speed. The adsorption mechanism of MB on the BMNC surface was evaluated through kinetics, adsorption isotherms, and thermodynamics. The experimental data indicated an endothermic, spontaneous, and thermodynamically favourable adsorption process, which was further supported by simulated modelling results using Forcite program. The in-silico data aligned well with the experimental findings. Additionally, the study assessed the interference of salts, metal ions, and other dyes on MB adsorption onto BMNC, showing promising results. These findings strongly support the effectiveness of our sorbent substrate under challenging conditions.

## Introduction

The presence of organic dyes in wastewater effluent from various industries, such as textile, leather, paper, plastic, cosmetics, and printing, poses a significant environmental challenge^1^. Approximately 10% of dyes used in dyeing processes are discharged into water bodies, leading to an increase in chemical oxygen demand and the disruption of aquatic ecosystems^2–4^. These organic dyes are not only environmentally hazardous but also have the potential to cause adverse health effects in humans. Methylene blue, a commonly used dye for cotton, timber, and silk dyeing, is known to be associated with health problems such as headaches, cancers, chest pain, and respiratory issues^5^.

Numerous scientific studies have explored various methods to remove or reduce dye content in wastewater^6^. These methods include flocculation and coagulation through chemical and physical processes, photo and biodegradation, oxidation using conventional and advanced techniques, nanotechnology-based filtration, and adsorption methods^7–9^. However, these methods often face limitations such as high operational costs, incomplete dye removal, and the generation of secondary products such as sludge, which may pose additional hazards compared to the initial dye pollutants. Therefore, there is a need to investigate effective techniques that can overcome the challenges associated with dye removal from colored wastewater.

Adsorption methods offer several advantages compared to other techniques, making them a favorable choice for dye removal. These methods are relatively low-cost, simple, and easy to manage in terms of both the removal and regeneration steps^10–12^. Factors such as operational cost, availability, toxicity, and reusability play a significant role in selecting the appropriate adsorbent. Various adsorbents have been reported in the literature for the removal of Methylene Blue (MB) dye.

Activated carbon materials with different morphologies have been widely studied for dye removal, but their high manufacturing cost limits their practical usage^13–16^. On the other hand, low-cost adsorbent materials such as fly ash^17^, modified cellulose^18,19^, agricultural wastes^20,21^, and clay minerals which can be used without chemical modification^22,23^, have shown promise in dye removal applications. Recent studies have demonstrated various natural clays, including montmorillonite^24,25^, bentonite^26–28^, sepiolite^29–31^, fibrous clay^32^, Kaolin^33,34^ and diatomite^35^, have been commonly used for the elimination of dyes from industrial effluents. By integrating these insights and referencing recent studies, our manuscript aims to contribute to the broader understanding of dye adsorption mechanisms and the development of efficient and sustainable adsorbents for wastewater treatment. In addition to experimental approaches, computational simulations, such as molecular dynamics (MD) simulations or density functional theory (DFT) calculations, can be employed to complement the experimental data. MD simulations provide atomistic-level insights into the interactions between MB molecules and clay nanoparticles, including orientation, diffusion, and binding energies. DFT calculations help understand the electronic structure and energetics of the adsorption process^36^. By comparing and validating computational results with experimental data, reliable models can be established to predict the adsorption mechanism of MB onto glauconite clay under different conditions or for different adsorbents. These predictions can be valuable for optimizing adsorption processes or designing more efficient adsorbents^37^.

The integration of experimental characterization, isotherm and kinetic modelling, and computational simulations provides a comprehensive understanding of the adsorption mechanism of methylene blue on BMNC. This combined approach contributes to the development of efficient adsorption processes for water treatment and environmental remediation applications^37^.

The primary purpose of this study is to explore the efficacy of nano-engineered glauconite clay (BMNC) as an innovative and sustainable adsorbent for the removal of methylene blue (MB) dye from polluted water. The significance of this work lies in the utilization of glauconite clay, sourced from the El Gidida region of Egypt, which has been subjected to nano-engineering to enhance its adsorption properties. This novel approach addresses the critical environmental issue of dye pollution, particularly from industries such as textiles and printing, which discharge large amounts of harmful dyes into water bodies. By optimizing the clay through nano-engineering, the study aims to increase the active surface area and improve the adsorption capacity of BMNC, thereby offering a cost-effective, efficient, and environmentally friendly solution for water purification. The integration of experimental results with computational insights further underscores the robustness of the adsorption mechanism, making this study a significant contribution to the field of environmental remediation and sustainable materials science.

## Materials and methods

### Nano glauconite preparation and characterization

Glauconite clay was obtained from the El Gidida area in Egypt. To prepare the nano glauconite (BMNC), the clay was milled in a laboratory-type ball mill for one hour to achieve a fine green product. The clay was mixed with hardened steel pellets in a vertical ball mill known as an "attritor". The attritor was made of stainless steel and had a water-cooled vessel. The milling process was carried out under an argon atmosphere at an operating speed of approximately 600 rpm with a clay-to-balls ratio of 1:20. After milling, the prepared clay was dried overnight in an oven at 100 °C. The final prepared clay (BMNC) was stored in a closed glass bottle.

The surface morphology and shape of BMNC were examined using an ultra-high-resolution transmission electron microscope (JEOL, JEM Instrument) operating at 200 kV. Scanning electron microscope (SEM) analysis coupled with energy-dispersive X-ray spectroscopy (EDS) was performed using a FESEM (Zeiss SEM Ultra) and an EDS instrument (Oxford Instruments) to obtain elemental composition information. X-ray fluorescence analysis (XRF) was conducted using a Burker S8 Tiger X-ray fluorescence spectrometer to measure the elemental composition of the clay.

The crystallinity features of BMNC were analysed using an X-ray diffraction (XRD) Siemens D5000 power diffractometer. Cu Kα radiation (λ = 0.15406 nm) with a Ni-filter was used, and the measurements were performed within the 2θ range of 10° to 100° with a scanning rate of 0.05°/s.

To determine the functional groups present on the surface of BMNC, Fourier-transform infrared (FTIR) analysis was conducted using an infrared spectrophotometer (Thermo Scientific Nicolet iS10 FTIR).

### Solutions preparation

A solution of Methylene Blue (MB) with a concentration of 10^3^ mg/L was prepared using MB powder of analytical grade (Merck) and distilled water. To prepare solutions with different MB concentrations, the concentrated solution was diluted with distilled water accordingly.

To obtain solutions with different pH levels, the pH of the MB solutions was adjusted using diluted solutions of NaOH (sodium hydroxide) and/or HCl (hydrochloric acid) from Merck. The pH adjustments were made as per the desired experimental conditions.

### Batch adsorption tests

The batch adsorption tests were conducted in 125 ml dark glass bottles using a lab shaking device at 200 rpm and a temperature of 25 °C. A total of 50 ml of diluted dye solution and a specified weight of BMNC were added to each bottle.

To study the effect of pH on the adsorption process, the pH of the prepared solutions was adjusted within the range of 2–9. This adjustment was achieved by adding dilute solutions of 0.1 M HCl or NaOH. The pH of the solutions was measured using a pH meter.

Different concentrations of MB solutions ranging from 10 to 400 mg/l were prepared to investigate the effect of MB concentration on the adsorption process. Additionally, varying masses of BMNC (ranging from 0.01 to 0.05 g) were used to study the effect of adsorbent dose. The experiments were conducted for different time durations, ranging from 5 to 480 min, to examine the effect of time on the adsorption process. Furthermore, the effect of temperature on the adsorption of MB was tested at temperatures ranging from 25 to 40 °C.

After the adsorption process, the remaining amount of MB in the solution was determined using a spectrophotometer technique (Shimadzu UV–visible, 1601PC, Japan) at a wavelength of 664 nm.

The adsorption capacity (Q_e_) for BMNC (mg/g) was calculated using Eq. (1):

1 $${Q }_{e}= \frac{\left({C}_{0} - {C}_{e}\right) V }{W}$$

where: Q_e_ (mg/g) is the adsorption capacity at equilibrium. C_o_ and C_e_ (mg/L) is the starting MB concentration and concentration at equilibrium, respectively. V is the solution volume (L). W is the adsorbent mass (g).

The removal efficiency was determined using Eq. (2):

2 $$Removal percent \%=\frac{({C}_{0} - {C}_{e})}{{C}_{0}}\times 100$$

### Determination of point zero charge (PZC)

To determine the point zero charge (PZC) of the ball-milled nano clay (BMNC), solutions with different pH values ranging from 2 to 11 were prepared using diluted solutions of HCl (0.1 M) and NaOH (0.1 M). Subsequently, 0.1 g of BMNC were mixed with 50 ml of each pH solution. After 24 h, the pH of each solution was measured.

## Results and discussion

### Characterization of BMNC sorbent

The XRF results were listed in Table 1. The obtained data showed that the main components of BMNC were composed of silica, iron, alumina, potassium, and magnesium. In Table 1, the term "LOI" stands for Loss on Ignition, which is a measure used to quantify the amount of volatile substances present in a material. LOI is determined by heating a sample to a high temperature (usually around 1000 °C) until all volatile substances are driven off, and then measuring the weight loss. This measurement provides an indication of the amount of organic matter, water content, carbonates, and other volatile compounds present in the sample. In the context of this study, the LOI value helps in understanding the composition and quality of the clay material used. Higher LOI values typically indicate higher amounts of organic and volatile components. For our ball-milled nano clay (BMNC), the LOI value is 10.5%, as shown in Table 1. This relatively high LOI value suggests a significant presence of organic matter and other volatile substances within the clay, which can affect its adsorption properties. The presence of these volatile components could enhance the adsorption capacity of BMNC by providing additional active sites for interaction with methylene blue dye molecules.

**Table 1:** The XRF analysis of the BMNC components.

Element	CaO	SiO_2_	Al_2_ O_3_	Fe_2_O_3_	MgO	SO_3_	
Percent	0.28	47.48	6.62	23.44	3.73	0.02	Total 99.99
Element	Na_2_O	K_2_O	P_2_O_5_	TiO_2_	MnO	LOI	
Percent	0.78	6.41	0.22	0.1	0.05	10.5	

**Fig. 1:**
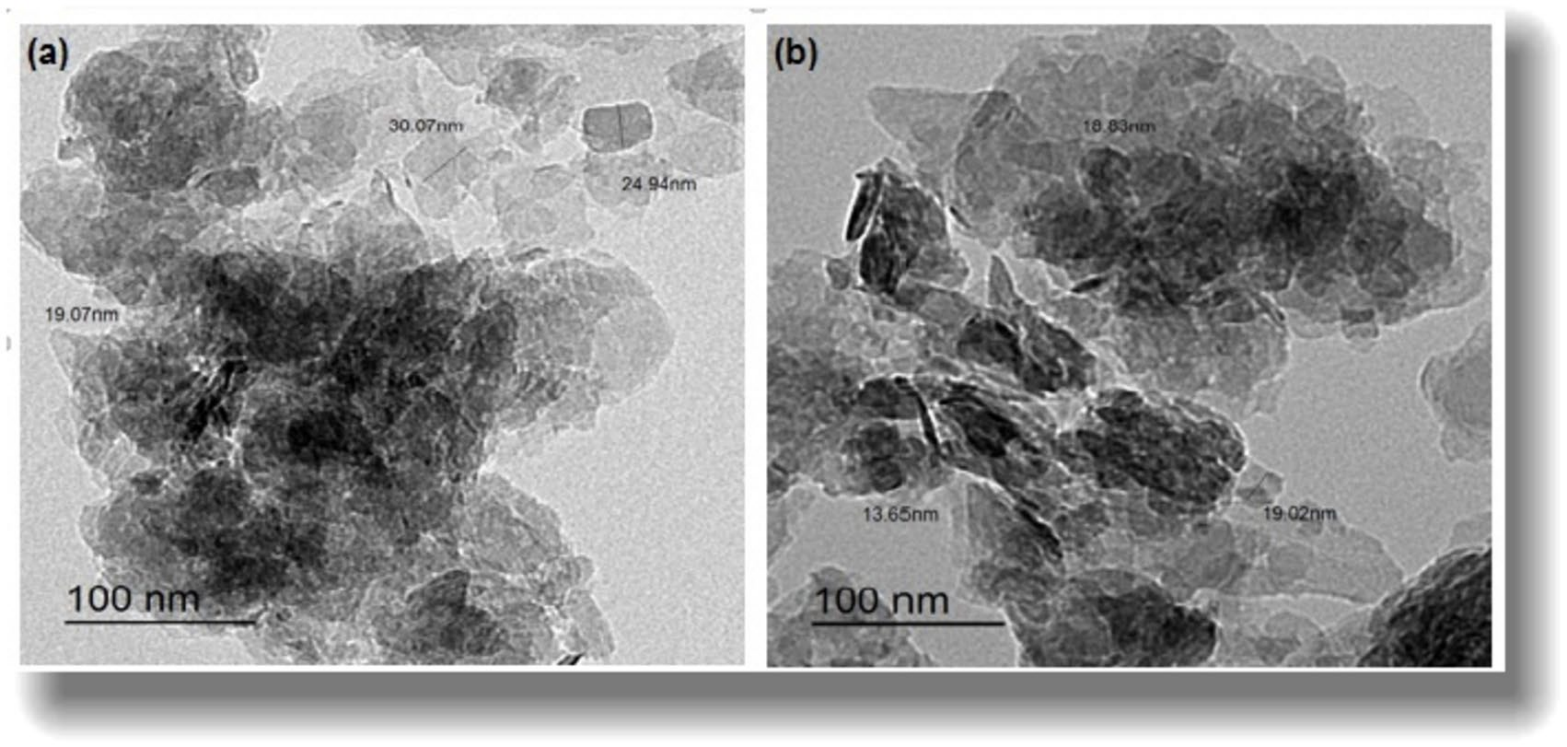
(**a**) TEM images of the free BMNC and (**b**) BMNC loaded by MB.

**Fig. 2:**
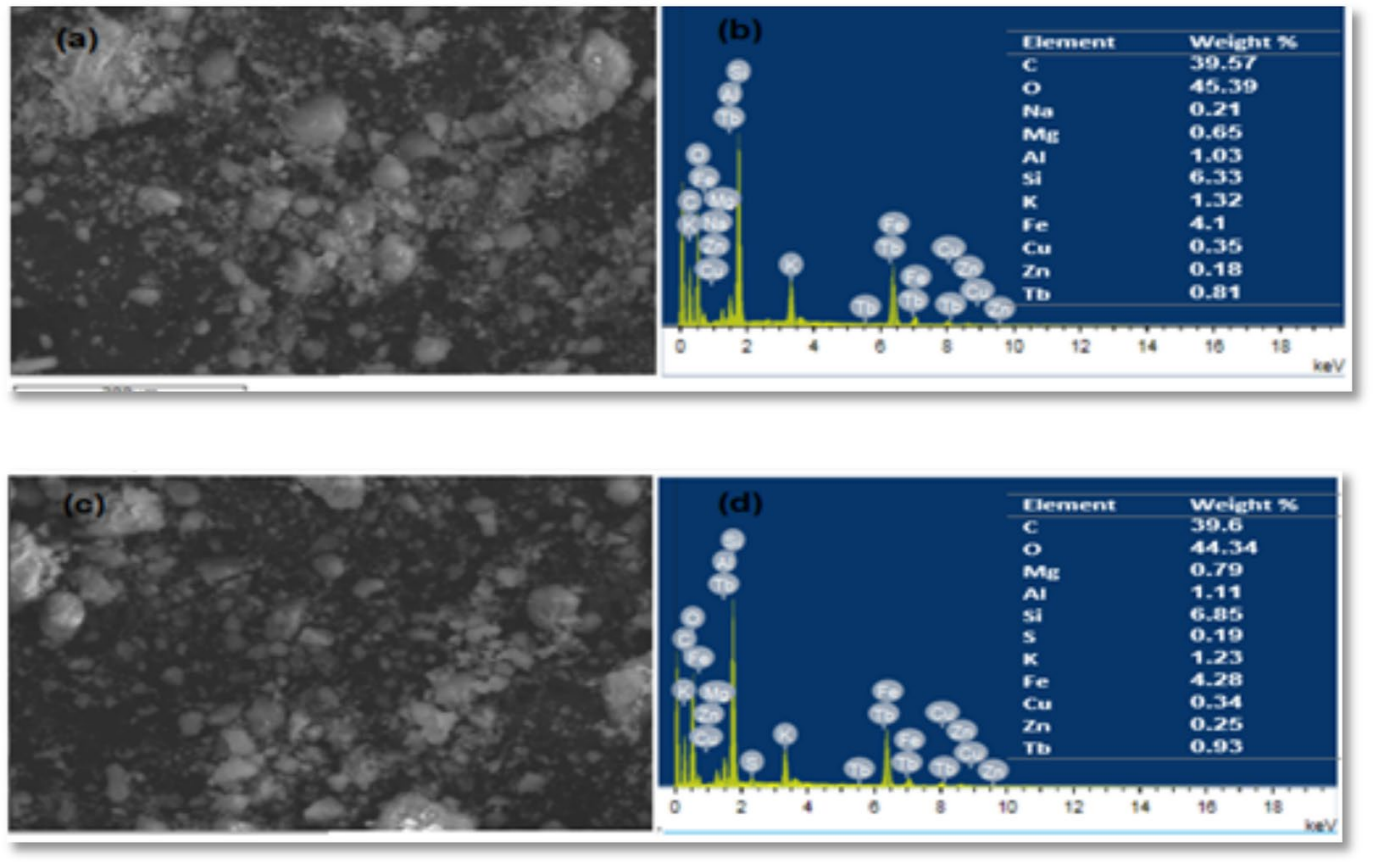
(**a**) The SEM pattern for BMNC, (**b**) the EDX results of the BMNC before adsorption process, (**c**) the SEM pattern for BMNC and (**d**) the EDX results of the BMNC after adsorption process respectively.

The TEM and SEM images of the BMNC are shown in Figs. 1 and 2, which illustrate that BMNC has an irregular structure and contains highly fractured pellets with altered rims, and the pellets consist of bent and curled crystals as highly crumped flakes in nano scale as well as decrease in particle size of loaded BMNC than the free BMNC (Fig. 1) which reveals the probability of the occupation of dye for the active sites at BMNC sorbent surface through ion exchange process^38^. Furthermore, the main elements found in the powder sample of sorbent before and after the sorption process by Energy Dispersive X-Ray Analysis (EDX) were viewed in Fig. 2. Solid sample after sorption has some elements not present in the sample before the sorption process, such as Sulfur, which belongs to MB dye and confirms the sorption process^39^ and the increase of carbon content after adsorption. Also, the reduction in sodium and potassium present reflects the possibilities of the ion exchange mechanism in this adsorption process.

**Fig. 3:**
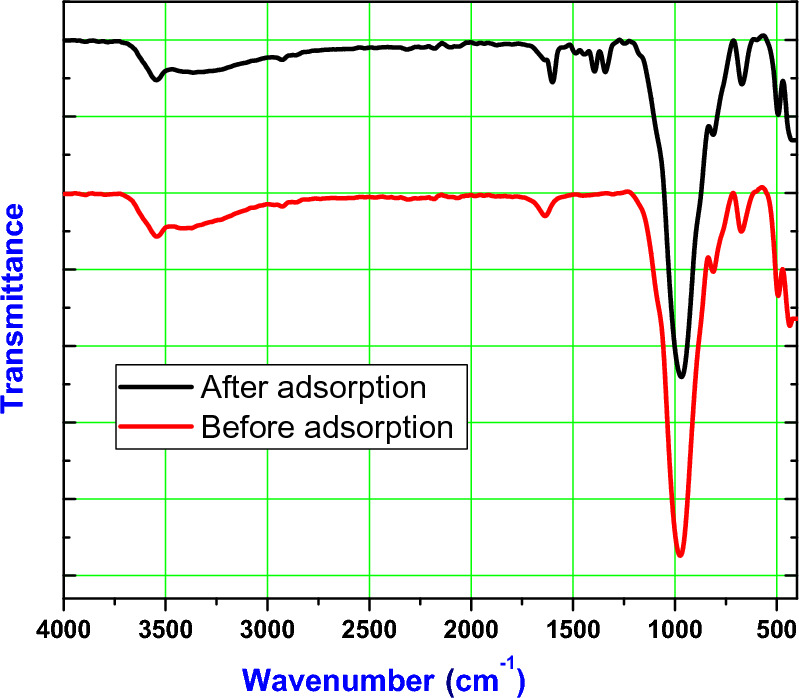
FTIR spectrum of the target BMNC before and dye adsorption.

The FTIR spectra of BMNC before and after dye removal is shown in Fig. 3. Broad peak corresponding to O–H stretching vibrations shifted slightly from 3543.6 to 3546.3 cm^−1^, indicating interaction of the hydroxyl groups with MB molecules, possibly through hydrogen bonding or complex formation^38^. The Si–O stretching vibrations peak at 1004 cm^−1^ before adsorption shifted to 1004.5 cm^−1^and showed a slight reduction in intensity, suggesting the involvement of silicate groups in the adsorption process. The peak corresponding to the OH group bending vibrations at 673.6 cm^−1^ before adsorption shifted to 670.8 cm^−1^ after adsorption, indicating interaction of OH groups with MB. The peak at 802.74 cm^−1^ showed a shift to 811.90 cm^−1^, suggesting the involvement of Al–OH groups in the adsorption mechanism. The metal–oxygen and metal-hydroxyl vibrations showed slight shifts in their respectively peaks, indicating interactions between MB molecules and metal–oxygen or metal-hydroxyl bonds. The H–O–H bending vibrations peak at 1624.06 cm^−1^ shifted to 1600.64 cm^−1^, which could be due to the interaction of water molecules with MB, forming hydrogen bonds^39^. The FTIR analysis before and after the adsorption of MB on BMNC indicates significant changes in the functional groups, confirming the interaction between the adsorbent and the adsorbate. These changes include shifts in the O–H, Si–O, Al–OH, and H–O–H vibrations, suggesting that multiple interactions such as hydrogen bonding, ion exchange, and complex formation are involved in the adsorption process. The detailed shifts in peak positions and changes in intensity provide valuable insights into the nature of these interactions.

The X-ray Diffraction (XRD) analysis provides essential information about the crystalline structure and phase composition of the ball-milled nano clay (BMNC) before and after the adsorption of methylene blue (MB). By comparing the XRD patterns, we can identify the structural changes that occur due to the adsorption process and gain insights into the interaction mechanisms between BMNC and MB.

**Fig. 4:**
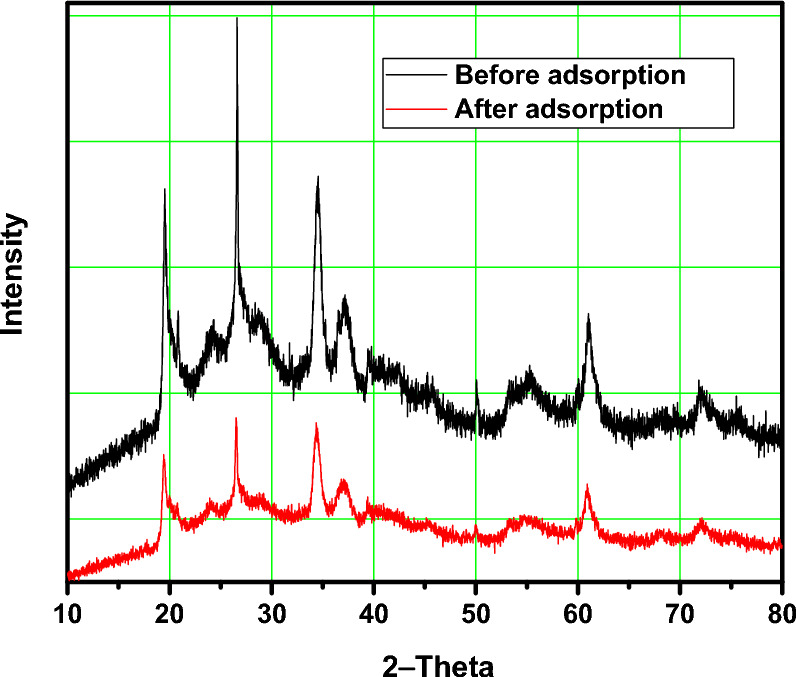
XRD patterns of the target BMNC before and dye adsorption.

XRD before and after the adsorption of MB, the XRD pattern of BMNC shows several characteristic peaks, indicating its crystalline nature (Fig. 4). The main peaks observed in the pattern include a prominent peak at 10.1° 2θ corresponding to the basal reflection of the layered clay structure which shifted slightly to a lower angle of 10.0° 2θ, suggesting an increase in the interlayer spacing due to the intercalation of MB molecules between the clay layers. A significant peak at 20.4° 2θ and 26.6° 2θ indicating the presence of silicate layers and quartz, respectively^40^. The intensity of the peaks at 20.4° 2θ and 26.6° 2θ decreased, indicating the adsorption of MB onto the clay surface, which affects the crystallinity of the silicate and quartz phases. A peak at 35.2° 2θ related to the presence of metal oxides and the peak at 50.1° 2θ, indicating the presence of crystalline phases within the clay. XRD after the adsorption of MB, notable changes were observed in the XRD pattern of BMNC. The XRD analysis before and after the adsorption of MB on BMNC indicates significant structural changes, confirming the interaction between the adsorbent and the adsorbate. The shifts in peak positions, reduction in intensity, and the appearance of new peaks provide valuable insights into the adsorption mechanism. The increase in interlayer spacing suggests intercalation of MB molecules, while the changes in intensity and new peak formation indicate the involvement of various phases in the adsorption process.

### Sorption factors

#### Effect of pH and evaluation of point of zero charge (PZC) for BMNC

**Fig. 5:**
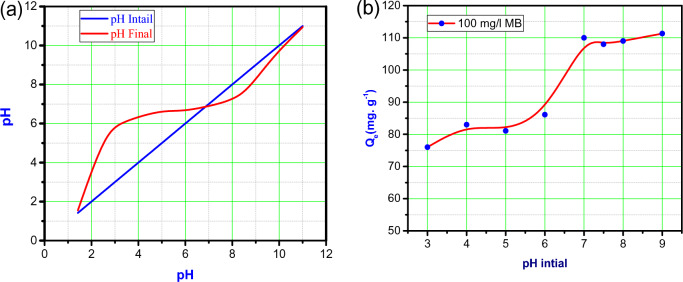
(**a**) The PZC for BMNC, (**b**) The effect of pH on dye sorption (100 mg/l) onto BMNC (dose was 0.025 g).

Based on the obtained pH measurements, a graph was plotted (Fig. 5a) to analyze the relationship between pH and the sorption capacity of BMNC. The graph indicated that the PZC of BMNC was found to be from 7 to 8. The PZC represents the pH value at which the net surface charge of the adsorbent is zero. This PZC value suggests that the previous pH range used (pH 7–8) is likely the optimum pH range for the adsorption of MB onto BMNC^41^.

Figure 5b illustrates the effect of pH on the adsorption process. It can be observed that at higher pH values (on the right side of the curve), the ability of BMNC to adsorb the dye increases. This is attributed to the increased negative charge on the clay surface, which enhances the electrostatic attraction between the negatively charged adsorbent surface and the cationic dye (MB). On the other hand, at lower pH values, the adsorption of MB decreases. This is because hydrogen ions occupy the active sites on the BMNC surface, hindering its ability to adsorb the dye. Additionally, at low pH, the adsorbent surface carries positive charges, leading to electrostatic repulsion between the positively charged adsorbent surface and the cationic dye^42^.

The highest adsorption capacity was observed within the pH range of 7–8, which is close to PZC of BMNC. This indicates that near the PZC, the surface of BMNC exhibits optimal charge characteristics for the adsorption of MB dye molecules.

#### Effect of time

**Fig. 6:**
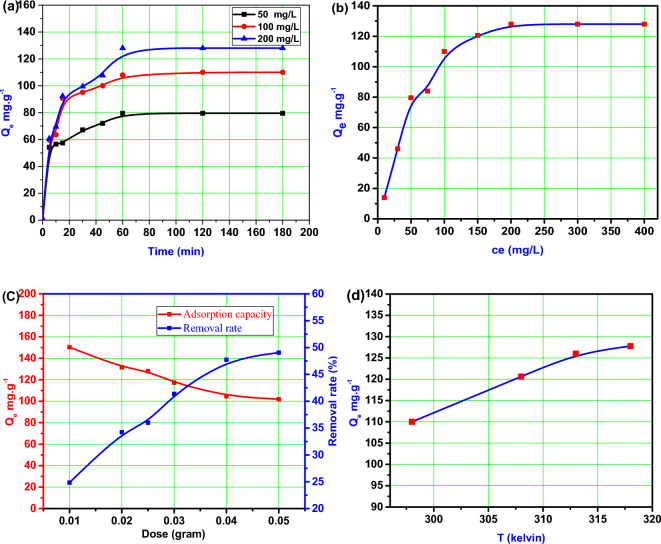
(**a**) Effect of time at pH = 7, dose 0.025 g and 50, 100 and 150 mg/l of MB dye, (**b**) effect of MB concentration at 0.025 g, pH = 7 and time = 60 min, (**c**) effect of substrate dose at pH = 7 and time = 60 min, 100 mg/l and (**d**) effect of temperature at 0.025 g, pH = 7 and time = 60 min and 100 mg/l on sorption of MB dye onto BMNC.

Figure 6a illustrates the effect of time on the adsorption capacity of BMNC for MB dye. The graph shows the adsorption capacity on the y-axis and the time in minutes on the x-axis. Three different concentrations of MB, namely 50 mg/l, 100 mg/l, and 200 mg/l, were used in the experiment.

The graph demonstrates that the adsorption of MB by BMNC is initially rapid within the first 30 min for all the MB concentrations tested. This rapid adsorption can be attributed to the larger number of available surface adsorption sites on BMNC during the initial stages of the process. After the initial 30 min, the adsorption rate gradually slows down. This reduction in adsorption rate can be attributed to a decrease in the number of active adsorption sites on the BMNC surface as they become occupied by MB molecules. At around 60 min, the adsorption capacity reaches its maximum for all the MB concentrations tested. This indicates that equilibrium has been reached, and no further active adsorption sites on BMNC are available to adsorb more MB from the solution. Beyond the 60-min mark, the MB concentration remains relatively constant, indicating that the adsorption process has reached a state of equilibrium^43,44^.

#### Effect of MB concentration

Figure 6b displays the effect of MB concentration on the adsorption capacity of BMNC after 60 min at pH 8. The graph shows the adsorption capacity on the y-axis and the MB concentration on the x-axis. The concentrations of MB ranged from 10 to 400 mg/l. The graph depicts that as the MB concentration increases from 10 to 200 mg/l, the adsorption capacity on the BMNC surface also increases. This indicates that the ability of BMNC to adsorb MB is enhanced with higher MB concentrations. The increase in adsorption capacity can be attributed to the greater driving force caused by the concentration gradient of MB between the solid phase (BMNC) and the liquid phase. However, after reaching an MB concentration of 200 mg/l, the adsorption capacity remains constant as the MB concentration is further increased to 400 mg/l. This suggests that there is a saturation point where the adsorption capacity of BMNC becomes independent of the MB concentration. The lack of further increase in adsorption capacity at higher MB concentrations can be attributed to the limited availability of vacant active sites on the BMNC surface to accommodate the excess MB molecules. As a result, the immediate solute adsorption becomes reduced due to the deficiency of vacant active sites required for the excessive MB concentrations. In summary, the adsorption capacity of BMNC for MB increases with increasing MB concentration up to a certain point, after which it reaches a plateau due to limitations in available active sites for adsorption^39,45^.

#### Effect of BMNC dose

Figure 6c presents the effect of BMNC dose on the adsorption process of MB. The adsorption experiments were conducted using various doses of BMNC ranging from 0.01 to 0.05 g, while keeping the MB concentration constant at 200 mg/l. The graph illustrates the impact of BMNC dose on both the adsorption rate of MB and the adsorption capacity. It is observed that as the dose of BMNC increases from 0.01 to 0.05 g, the adsorption capacity of MB decreases from 150 to 102 mg/g. This decrease in adsorption capacity can be attributed to the presence of residual unsaturated sites on BMNC for higher doses. In other words, as the dose of BMNC increases, a larger amount of clay is available for adsorption, but not all of the adsorption sites are utilized, leading to a lower overall adsorption capacity^26^. On the other hand, the removal rate of MB increases from 25 to 50% with the increase in BMNC dose from 0.01 to 0.05 g. This higher removal rate can be attributed to the increase in the surface area and the number of available adsorption sites on BMNC. With a higher BMNC dose, there is a larger surface area for MB molecules to interact with, resulting in a more efficient adsorption process and higher removal rates^46^. In summary, increasing the dose of BMNC leads to a decrease in the adsorption capacity of MB due to unsaturated adsorption sites. However, it also results in higher removal rates of MB, attributed to the increased surface area and availability of adsorption sites on BMNC.

#### Effect of temperature

Figure 6d depicts the effect of temperature on the adsorption capacity of MB onto BMNC. The experiments were conducted using temperatures ranging from 25 to 45 °C, with an initial MB concentration of 100 mg/l and a fixed BMNC dose of 0.025 g. The graph shows that as the temperature increases from 25 to 45 °C, the adsorption capacity of MB onto BMNC also increases^47^.

The adsorption capacity rises from 110 mg/g at 25 °C to 127 mg/g at 45 °C. This increase suggests that the adsorption process involves both physical and chemical sorption mechanisms. The rise in adsorption capacity with increasing temperature indicates that the adsorption process is endothermic, meaning it is favored by higher temperatures. The increased thermal energy at higher temperatures enhances the adsorption process by facilitating the interactions between the MB molecules and the BMNC surface. This can lead to improved surface coverage and stronger adsorption of MB onto BMNC. In summary, the increase in temperature from 25 to 45 °C enhances the adsorption capacity of MB onto BMNC, implying the involvement of physical and chemical sorption processes. The endothermic nature of the adsorption process suggests that higher temperatures favor the adsorption of MB onto BMNC^48^.

### Adsorption kinetics

The adsorption mechanism of MB onto BMNC was investigated using three kinetic models: the pseudo-first-order (PFO) model, the pseudo-second-order (PSO) model, and the intra-particle diffusion model. These models were applied to the experimental data to understand the kinetics of the adsorption process.

#### Pseudo-1st-order model (PFO)

**Fig. 7:**
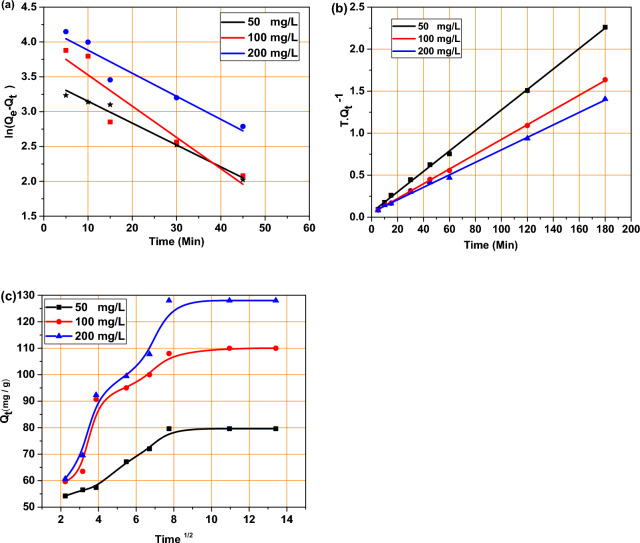
(**a**) Pseudo-1st-order model, (**b**) Pseudo-2nd-order model, (**c**) intra-particle diffusion model, for MB sorption onto BMNC (dose was 0.025 g).

According to the information provided, the pseudo-first-order (PFO) model was applied to the adsorption of MB onto BMNC. The PFO model is represented by linear Eq. (3) and non-linear Eq. (4), where Q_e_ and Q_t_ are the amounts of MB adsorbed (mg/g) at equilibrium and at time t, respectively^49^. The rate constant of the PFO model is denoted as k_1_ (min^−1^). The linear plots of log(Q_e_ − Q_t_) versus t (Fig. 7a) show the fitting of the pseudo-first-order model. The calculated values of Q_e_ from the linear regression deviate significantly from the experimental values, indicating poor fitting. The correlation coefficients (R^2^) were relatively low, suggesting that the pseudo-first-order model is not adequate for describing the adsorption kinetics of methylene blue (MB) onto BMNC. However, it is mentioned that the calculated values of Q_e_ (31.9, 53.23, and 67.49 mg/g) for MB concentrations of 50, 100, and 200 mg/l, respectively, deviate from the experimental values (Table 2). In the non-linear regression, the model parameters were adjusted to minimize the difference between experimental and predicted values (Supplementary Fig. S1). However, the non-linear fitting still showed significant deviation, confirming that this model does not suitably describe the adsorption process. This indicates that The PFO model is not an appropriate model to explain the adsorption mechanism of MB on BMNC.

3 $$\mathit{log}\left({Q}_{e}- {Q}_{t}\right)=\mathit{log}{Q}_{e}- \frac{{k}_{1}}{2.303 }t Linear$$

4 $${Q}_{t}={Q}_{e}\left(1-\mathit{exp}\left(-{K}_{1}t\right)\right) Nonlinear$$

where Q_e_ is the amount of adsorbate adsorbed at equilibrium (mg/g), Q_t_ is the amount of adsorbate adsorbed at time t (mg/g) and k_1_ is the rate constant of the pseudo-first-order adsorption (1/min).

**Table 2:** Adsorption kinetics parameters for the target models.

Concentration (mg/L)		50		100		200	
Q_e (exp)_ (mg/g)		81		110		128	
	Parameters	Linear	Non-linear	Linear	Non-linear	Linear	Non-linear
Pseudo-first-order	Q_e (cal)_ (mg/g)	31.92	73.9	53.24	104.4	67.5	117.8
	k_1_ (1/min)	0.031	0.18	0.045	0.12	0.033	0.1017
	R^2^	0.984	0.5215	0.873	0.87	0.924	0.87
Pseudo-second-order	Q_e (cal)_ (mg/g)	82.03	79.2	113.87	113	134.58	129.6
	k_2_ (g/mg min)	0.0026	0.00371	0.0019	0.0017	0.0011	0.00111
	R^2^	0.999	0.820	0.999	0.920	0.998	0.950
Intraparticle diffusion	k_p_ (mg/g min^1/2^)	–	2.566	–	4.12	–	5.593
	C	–	51.078	–	64.009	–	62.767
	R^2^	–	0.819	–	0.667	–	0.769

#### Pseudo-2nd-order model (PSO)

The pseudo-second-order (PSO) model was applied to the adsorption of MB onto BMNC. The pseudo-second-order (PSO) model described by linear Eq. (5) and non-linear Eq. (6). The linear plots of t/Qt versus t (Fig. 7b) show a much better fit for the pseudo-second-order model. The correlation coefficients (R^2^) were very high (close to 0.999), and the calculated values of Q_e_ were very close to the experimental values, indicating that the pseudo-second-order model accurately describes the adsorption kinetics. In the non-linear regression, the pseudo-second-order model provided an excellent fit, with high R^2^values and minimized error (Supplementary Fig. S1). This suggests that the adsorption process is likely controlled by chemisorption involving valence forces through sharing or exchange of electrons between adsorbent and adsorbate^44,51^. Table 2 provides the calculated values of Q_e_ and R^2^ for MB concentrations of 50, 100, and 200 mg/l. It is stated that all correlation coefficients for these concentrations are higher than the results obtained from the PFO model.

The PSO model is represented by linear Eq. (5) and non-linear Eq. (6), where k_2_ is the rate constant of The PSO (mg/g min^−1^)^50,51^. In Fig. 6b, a plot was generated between t/Q_t_ and time (t) using the experimental data. The intercept and slope of this plot can be used to determine the values of Q_e_, k_2_, and the correlation coefficient (R^2^). Table 2 provides the calculated values of Q_e_ and R^2^ for MB concentrations of 50, 100, and 200 mg/l. It is stated that all correlation coefficients for these concentrations are higher than the results obtained from The PFO. The correlation coefficients are reported as 0.998, 0.999, and 0.997 for 50, 100, and 200 mg/l, respectively. Additionally, it is mentioned that the calculated values of Q_e_ using the PSO model are closer to the experimental values compared to the PSO model. This suggests that the kinetics of MB adsorption onto BMNC are better represented by the PSO model. Based on these results, it can be concluded that The PSO model, which involves chemisorption and valence forces through ion sharing exchange between BMNC and MB, effectively describes the adsorption kinetics of MB onto BMNC^45,52^.

5 $$\frac{t }{{Q}_{t}} = \frac{1 }{{k}_{2}{Q}_{e}^{2}} + \frac{t }{{Q}_{e}} Linear$$

6 $${Q}_{t}=\frac{{Q}_{e}^{2} {K}_{2}t}{1+{Q}_{e}{K}_{2}t} Nonlinear$$

#### Intra-particle diffusion model

According to the provided information, the intra-particle diffusion model was applied to analyze the adsorption of MB onto BMNC. The intra-particle diffusion model is represented by Eq. (5), where k_d_ is the intra-particle diffusion rate constant (mg/g min^−1/2^), t^1/2^ represents the square root of time, and C is the intercept that represents the boundary layer thickness^41^. In Fig. 7c, a linear plot was generated between Q_t_ and t^1/2^ using the experimental data. The intercept and slope of this plot can be used to determine the values of C and k_p_, respectively. Table 2 provides the calculated values of C and k_p_, as well as the corresponding R^2^ values. It is mentioned that the calculated R^2^ values for the intra-particle diffusion model are low, indicating that this model does not adequately describe the adsorption process of MB onto BMNC. Although the intra-particle diffusion process may play a role in the adsorption, there are likely better models available to explain the adsorption mechanism. Therefore, based on these results, it can be concluded that while the intra-particle diffusion process contributes to the adsorption of MB onto BMNC, the low R^2^ values suggest that alternative models should be explored to better describe the adsorption process.

7 $${Q}_{t} = {k}_{p}{t}^{1/2}+C$$

where: kp is the intra-particle diffusion rate constant (mg/g·min0.5) and C is the intercept which indicates the boundary layer effect. The non-linear form is more complex and is often represented by fitting the data directly to the empirical equation.

### The adsorption isotherm

To understand the interaction and binding mechanism for adsorption of MB onto the target clay, several of adsorption isotherms were used and the results of those isotherms models gave some idea about the different adsorption capacities and play a significant role in identifying the probable mechanisms for adsorption^53^. In this work, Langmuir, Freundlich, Temkin, and Dubinin–Radushkevich (D–R) have been applied for the adsorption process. The outcomes of these models are estimated and compared not only for correlation coefficient R^2^ but also for adsorption capacities.

Langmuir isotherm model based on unimolecular combination form of adsorption process. The Langmuir model were represented by linear Eq. (8) and nonlinear Eq. (9)^54,55^.

8 $$\frac{{C}_{e}}{{Q}_{e}}=\frac{1}{{{K}_{1}Q}_{m}}+\frac{{C}_{e}}{{Q}_{m}}$$

9 $${Q}_{e}={Q}_{m}\frac{{K}_{1}{C}_{e}}{1+{K}_{1}{C}_{e}}$$

**Fig. 8:**
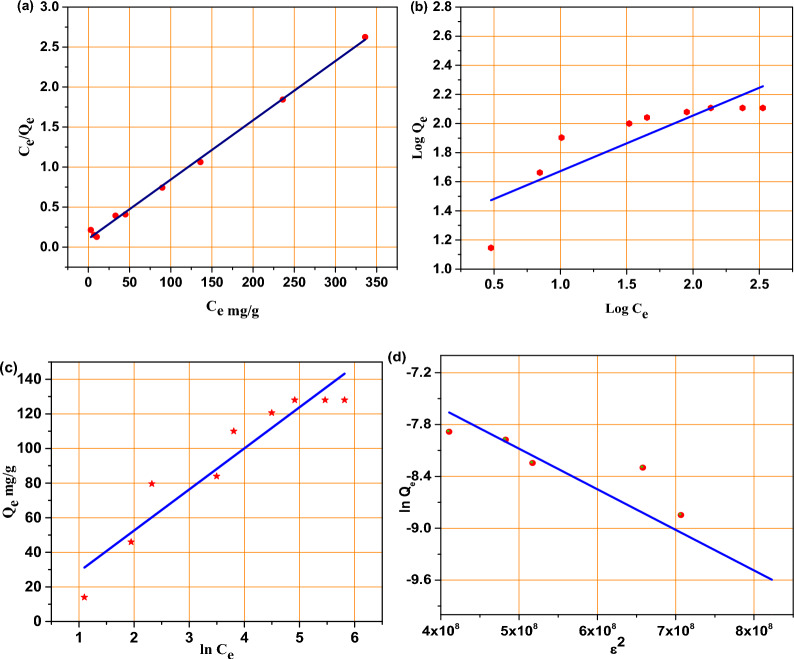
(**a**) The Langmuir linear isotherm, (**b**) Freundlich linear isotherm, (**c**) Temkin linear isotherm and (**d**) Dubinin–Radushkevich linear isotherm models, (**e**) non-linear form of isotherm models.

Q_m_ and Q_e_ are the theoretical maximum adsorption capacity (mg/g) and the adsorption capacity at equilibrium (mg/g), respectively, while b is representing the Langmuir adsorption constant (L/mg). C_e_ is the remaining concentration of MB in the solution (mg/l). The obtained straight-line plot of C_e_/Q_e_ versus C_e_ in Fig. 8a indicate that the high probability for adsorption process of MB by BMNC to follows the Langmuir isotherm model where R^2^ equal to 0.9971. Langmuir constants were calculated and listed in Table 3. The calculated Q max was 135 mg/g which nearly closed to the experimental adsorption capacity (128 mg/g). The following equation was used to calculate very important parameter for Langmuir isotherm using Langmuir constant and the initial concentration of MB (${\text{C}}_{0}$).

**Table 3:** Isotherm parameters for adsorption of MB dye by BMNC adsorbent.

Isotherm	Parameters	Linear	Non-linear
Langmuir	Q_m_ (mg/g)	135	135
	b (L/mg)	0.07	0.0817
	R_L_	0.033–0.58	0.03–0.55
	R^2^	0.997	0.944
Freundlich	1/n	2.62	4.19
	K_F_ (L/mg)	12.3	36.4
	R^2^	0.726	0.808
Temkin	B1	1.24	1.24
	K_t_ (L/mg)	23.7	23.74
	R2	0.902	0.902
D–R	Q_s_ (mg/g)	290	118.6
	B (mmol^2^/kJ^2^)	4.7 × 10^–9^	0.02076
	R_2_	0.907	0.9
	E(KJ/mole)	10.3	4.9

In the non-linear regression, the Langmuir model provided an excellent fit with minimized error values and high R^2^ values, further confirming the monolayer adsorption hypothesis (Supplementary Fig. S2).

10 $${R}_{L}=\frac{1}{\left(1+{K}_{1}{C}_{0}\right)}$$

where R_L_ represented dimensionless separation factor of the equilibrium parameter.

The calculated R_L_ value could be used to categorize the isotherm type either unfavorable (R_L_ > 1), linear (R_L_ = 1), favorable (0 < R_L_ < 1), or irreversible (R_L_ = 0).

In this work, the results are indicating that adsorption process is favorable as the values of R_L_ value were found in the range of 0.0339–0.584 for the concentrations range 400–10 mg/l^56^.

Freundlich isotherm model were represented by linear Eq. (11) and nonlinear Eq. (12)^57^.

11 $$\mathit{log}{Q}_{e}=\mathit{log}{K}_{f}+\frac{1}{n} log {C}_{e}$$

12 $${Q}_{e}={K}_{f}{C}_{e}^{1/n}$$

K_F_ and n are the Freundlich capacity constant (L/mg) and the Freundlich intensity constant respectively. Figure 8b represents a plot of log Q_e_ versus log C_e_ which owned non good linearity properties that reflect the very low probability of the adsorption process to follow this isotherm assumption. The calculated parameters of freundlish isotherm are listed in Table 3. The slope value was 0.38 is closer to zero that reflect the heterogeneous adsorption process^58^. From Table 3 adsorption process was indicated to the high probability to follow the Langmuir isotherm model than Freundlich adsorption isotherm as Langmuir produced a better fit for the experimental equilibrium adsorption data than the Freundlich model.

The non-linear regression of the Freundlich model showed a slightly better fit with higher R^2^ value compared to Langmuir model, suggesting that the Freundlich model can not describe the adsorption process for heterogeneous surfaces (Supplementary Fig. S2).

The Temkin isotherm assumed that the heat of adsorption reduced linearly with increasing in coverage of adsorbent by the adsorbate. the Temkin model were represented by linear Eq. (13) and nonlinear Eq. (14)^59^.

13 $${\text{Q}}_{\text{e}}={\text{B}}_{1 }\text{ln }{\text{K}}_{\text{t}}+{\text{B}}_{1 }{\text{lnC}}_{\text{e}}$$

14 $${Q}_{e}={B}_{1}\text{ ln}({K}_{t}{C}_{e})$$

Q_e_ represent the dye amount adsorbed at equilibrium; B_1_ and K_t_ are the Temkin adsorption heat constant and the Temkin isotherm equilibrium binding constant, respectively. The slope and intercept calculated from the Q_e_ versus lnC_e_ graph in Fig. 8c, used to calculate Temkin energy constants. All Temkin parameters calculated and listed in Table 3. The R^2^ value was 0.902, which presents a weak fitting of the Temkin isotherm model to illustrate this adsorption process.

In the non-linear regression, the Temkin model provided a reasonable fit with improved R^2^ values, indicating that the model can describe the adsorption process considering adsorbate/adsorbate interactions (Supplementary Fig. S2).

The Dubinin–Radushkevich isotherm (D–R) is usually applied to definite the mechanism for studied adsorption experiments and evaluate porosity properties for adsorbent surface as well as the apparent energy of adsorption. The (D–R) isotherm model were represented by linear Eq. (15) and nonlinear Eq. (16)^60^:

15 $$\text{ln }{\text{Q}}_{\text{e}} =\text{ ln }{\text{Q}}_{\text{m}}-\left(B{\upvarepsilon }^{2}\right)$$

16 $${Q}_{e}={Q}_{m}exp(-B{\upvarepsilon }^{2})$$

where Q_m_ represent the theoretical adsorption capacity (mol/g), B represent (D–R) isotherm constant that related for the adsorption mean free energy per mole of adsorbate (mmol^2^/J^2^), and $\upvarepsilon$ is the Polanyi potential represent equilibrium and calculated according to Eq. (17).

17 $$\upvarepsilon =\text{ RT ln }\left(1 + \frac{1 }{{\text{C}}_{\text{e}}}\right) $$

where R represent the universal gas constant equal to 8.314 J mol^−1^ K^−1^ and T represent the temperature in Kelvin unit. From Fig. 8d by plotting lnQ_e_ versus ε^2^, a straight line was obtained with a correlation coefficient = 0.907, a slope equal to K_d_, and an intercept equal to lnQ_s_. The Q_s_ and B values were calculated from the slope and intercept and found to be 290 (mg/g) and 4.7 × 10^–9^ (mmol^2^/kJ^2^), respectively. From Eq. (18), the mean adsorption energy was calculated and gave 10.3 kJ/mol, which reflects the ion exchange explanation for this adsorption process^61^.

18 $$\text{E}= \frac{ 1 }{\sqrt{2\text{B}}}$$

However, the resulting R^2^ is not the highest value among the applied isotherms models, which indicates poor fitting to this isotherm model. The outcomes of this study approve that the Langmuir isotherm model is the most suitable isotherm to illustrate the adsorption of MB dye by BMNC adsorbent.

The non-linear regression of the D–R model provided a good fit with high R^2^ values, supporting the conclusion that the adsorption mechanism is primarily ion exchange with some ion exchange characteristics (Supplementary Fig. S2).

The comparison of linear and non-linear regression results for the isotherm models indicates that the Langmuir model provides the best fit for the adsorption data, suggesting monolayer adsorption on a homogeneous surface. The Freundlich and Temkin models also show reasonable fits, indicating adsorption on heterogeneous surfaces and considering adsorbate/adsorbate interactions. The D–R model supports the conclusion that the adsorption process involves physical mechanisms with some ion exchange characteristics. By providing detailed analysis and interpretation of both linear and non-linear models, we can better understand the adsorption mechanisms and capacities, leading to more accurate and reliable predictions of the adsorption process.

### Thermodynamics of adsorption

With the purpose of better empathizing how the temperature effects on the adsorption process of MB on BMNC, the following basic thermodynamic parameters were examined: Gibbs free energy of adsorption ($\Delta \text{G}^\circ$), Enthalpy change (∆H$^\circ$), and Entropy change (∆S$^\circ$). From the Eq. (19), using the equilibrium constant ${K}_{C}^{^\circ }$, the Gibbs free energy of the adsorption was evaluated^62^.

19 $$\Delta \text{G}^\circ =-\text{RT In }{\text{K}}_{\text{C}}^{^\circ }$$

where R represent the universal gas constant (8.314 J/mol K) while T represent the experiment temperature in Kelvin. The following equation was used to get the value for thermodynamic adsorption equilibrium constant, K_C_°.

20 $${\text{K}}_{\text{C}}^\circ =\frac{{\text{C}}_{\text{a}}}{{\text{C}}_{\text{e}}} $$

C_a_ and C_e_ represent adsorbed MB dye onto BMNC (mg/l) and the remaining MB dye in the solution (mg/l) respectively. Then Enthalpy changes as well as entropy change were calculated using the below equation^63^:

21 $$\Delta \text{G}^\circ =\Delta \text{H}^\circ -\text{T}\Delta \text{S}^\circ = -\text{RT In }{\text{K}}_{\text{C}}^{^\circ }$$

22 $$\text{In }{\text{K}}_{\text{C}}^{^\circ } = \frac{\Delta \text{S}^\circ }{\text{R}}- \frac{\Delta \text{H}^\circ }{\text{RT}}$$

**Fig. 9:**
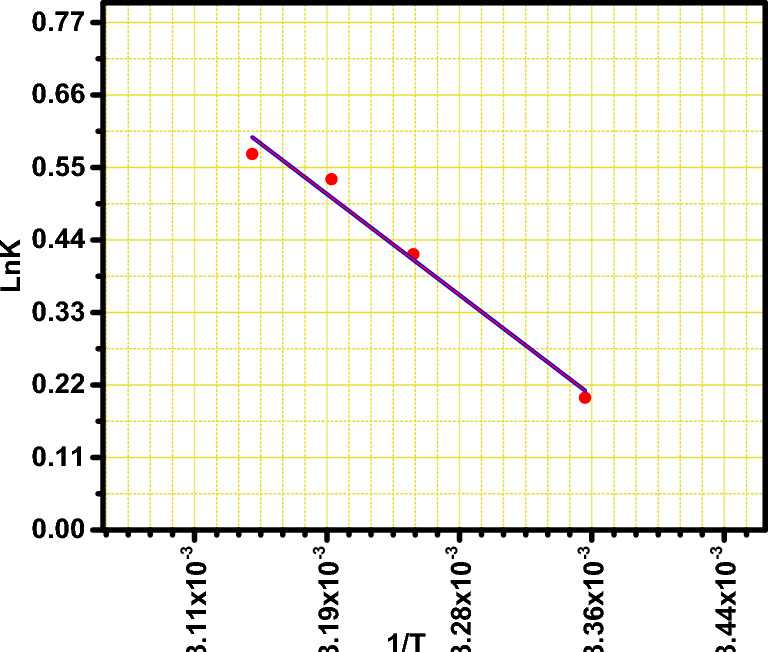
Van’t Hoff plot for the adsorption of MB (100 mg/l) onto BMNC (0.025 g).

From the slope and intercept of the plots of lnK_c_ against T^−1^ (Van't Hoff plot), the values of ∆H$^\circ$ and ∆S$^\circ$ were obtained as shown in the Fig. 9.

The obtained thermodynamic parameters were listed in Table 4. The negative values of the ∆G° revealing the spontaneous nature of the adsorption process in addition to the thermodynamically favorable behavior. The adsorption process can be categorized according to the degree of the enthalpy change into physisorption and chemisorption^18,55^.

**Table 4:** Thermodynamics parameter for the adsorption of the MB on the target clay.

Temp(k)	∆G*°* (kJ/mol)	∆H*°* (kJ/mol)	∆S*°* (J/mol K)
298	− 0.497	15.1	53
308	− 1.071		
313	− 1.385		
318	− 1.508		

The positive values of ∆H^o^ emphasize the endothermic and physical nature of the studied adsorption process; therefore, with rising temperature, the adsorbed amount of dye at equilibrium increased. Besides, the increase in the degrees of freedom of the solid–liquid interface during the adsorption process were confirmed as result of positive ∆S^o^ which suggested the ion-exchange adsorption mechanism^48,64^.

### Effect of ionic strength

**Fig. 10:**
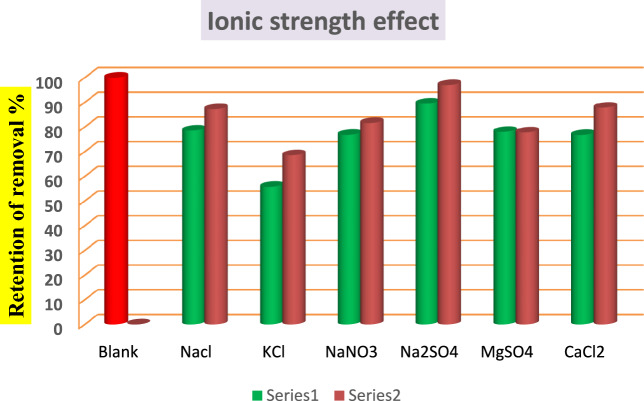
Effect of ionic strength on the adsorption of MB onto BMNC sorbent. Series 1 = 0.1 N. Series 2 = 0.05 N.

To study the influence of ionic strength on the methylene blue sorption by BMNC, several sorption trials were evaluated by two different sets of concentrations (0.05 M and 0.1 M) for the following salts: NaCl, NaNO_3_, KCl, Na_2_SO_4_, CaCl_2_, and MgSO_4_ (Merck) with 50 ml from 100 mg/l MB dye and 0.025 g glauconite for every salt. From Fig. 10, it is clear that the existence of salts at any concentration affected the adsorption process by decreasing the adsorption capacity of BMNC for MB dye. Besides, increasing the ionic strength decreased the dye adsorbed at equilibrium. These results can be as result of the surface of BMNC becomes not easily accessible for methylene blue adsorption due to the competition of salts ions toward the active sites on the glauconite surface^24^. Furthermore, the cation with a smaller hydrated radius would occupy more functional sites on the glauconite surface as it could difficulty escape from the adsorbent surface^65^. Since the hydrated radius of K^+^ is the smallest among other cations of used salts, causing a stronger competition with MB^+^, potassium ion generates a clearer effect on MB adsorption compared with the other cations. So, the lower adsorption capacity of MB was found when competed with KCl matrix.

### interferences study

To investigate the effect of some heavy metal ions and other dyes on the MB dye adsorption process onto the BMNC, some sorption trials experimented with a concentration of 100 mg/l from Lead, Copper, Nickel, and Cobalt with 50 ml from 100 ppm MB dye and 0.025 g glauconite for heavy metal interferences and 100 mg/l from Malachite green (MG) and Rhodamine B (RB) with 50 ml from 100 mg/l MB dye and 0.025 g glauconite for dyes interferences.

**Fig. 11:**
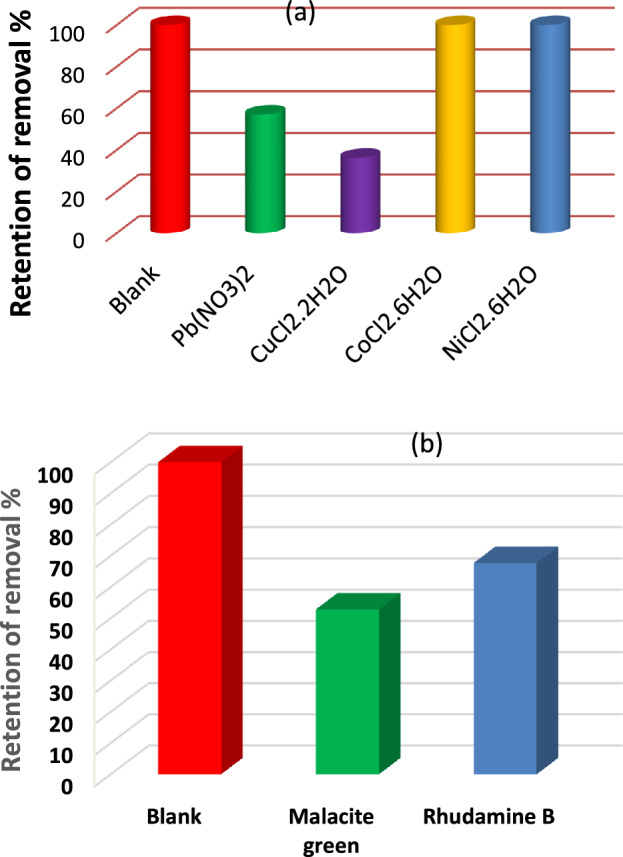
(**a**) Effect of heavy metals and (**b**) effect of other dyes on adsorption of MB onto BMNC sorbent.

From Fig. 11a, it is clear that Lead and Copper hindered MB sorption, causing a sharp shrink in adsorption capacity, revealing the affinity of these metals to the BMNC surface. Moreover, BMNC was used for removing Lead and Copper by the adsorption process^66–68^.

From Fig. 11b, it is clear that MG and RB competent MB sorption, revealing the affinity of these cationic dyes to the BMNC surface. Moreover, BMNC was used for removing cationic dyes' adsorption process^69^.

### In silico studies and computational chemistry approach

#### Simulation approach using the EXPO program

**Fig. 12:**
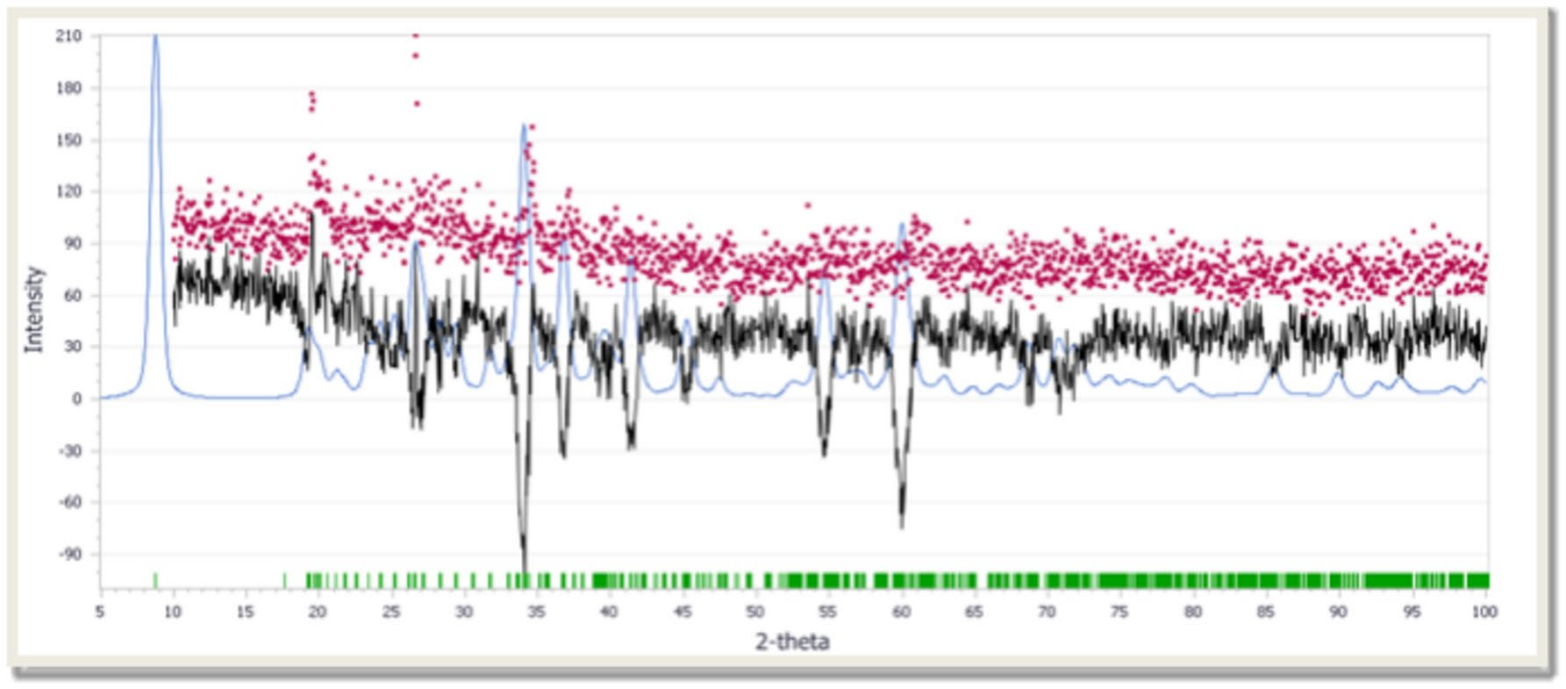
The calculated (blue), measured (red), difference (black) and observed reflection (green) for the target biotite mimic.

The EXPO program can perform all the steps of the structure solution process by powder diffraction data: indexing, space group determination, estimation of the reflection integrated intensities, structure solution by direct methods or/and by direct space/hybrid approaches, model refinement by Rietveld technique. The program has been designed to: require minimal information as input, work automatically, reduce user intervention, and facilitate interaction through a user-friendly graphic interface^70^. XRD analysis for the target material was measured, and the calculated cell parameters were evaluated using the EXPO2014 program^71^. The obtained calculated results go well with the experimental data as displayed in Fig. 12. The calculated cell parameters are displayed in supplementary data (Supplementary Table S1). The obtained crystal system was monoclinic with Patterson space group = P2/m. The dimension and the angles of the simulated crystal are recorded in supplementary data (Supplementary Table S1).

#### Energy optimization of the MB dye

The methylene blue dye was listed and optimized its geometry by Forcite tools. Forcite is a collection of molecular mechanics tools that allow you to investigate various systems. The key approximation is that the potential energy surface on which a classical force field represents the atomic nuclei moves. Force fields are developed by parameterizing data from experiments and high-level quantum mechanical calculations. The COMPASS force fields were used in our work. Forcite is used to optimize the geometry of a system prior to a molecular dynamics simulation or a quantum mechanical calculation. The view of the optimized geometry well showed in supplementary data (Supplementary Fig. S3). The optimized geometry energy = 49.73 kcal/mol^72^.

#### Geometric optimization of the target layer silica material

The target silica material from the layer type has a molecular formula (K, Na)(Fe, Al, Mg)_2_(Si, Al)_4_O_10_(OH)_2,_ which mimics the biotite family. The XRD analysis and the calculated cell parameters could present the crystal structure of the target compound, as shown in supplementary data (Supplementary Fig. S4). Si and Al have tetrahedral structures. Meanwhile, Fe has an octahedral structure. The Forcite energy of the optimized geometry = 115.32 kcal/mol^72^.

#### Geometric optimization of the adsorption of MB dye on BMNC

**Fig. 13:**
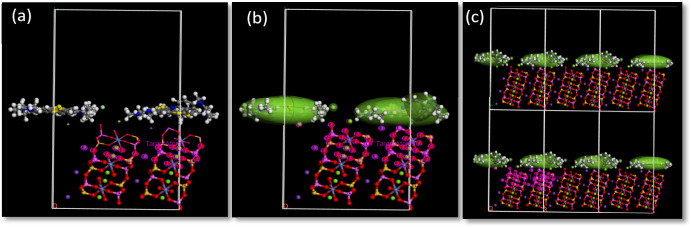
(**a**) The adsorption system in xz plane free molecule, (**b**) ellipsoid form and (**c**) the overall adsorption system.

The adsorption process of the dye molecules (methylene blue) on the surface of the target moiety BMNC was evaluated using adsorption locator tools. The adsorption locator enables the simulation of a substrate loaded with an adsorbate or an adsorbate mixture of a fixed composition. The adsorption locator is designed to study individual systems, allowing us to find low-energy adsorption sites on periodic and non-periodic substrates or investigate the preferential adsorption of mixtures of adsorbate components. Adsorbates are typically molecular gases or liquids, and substrates are usually porous crystals or surfaces, such as zeolites or carbon nanotubes, or amorphous structures, such as silica gel or activated carbon. The adsorption locator identifies possible adsorption configurations by carrying out Monte Carlo searches of the configurationally space of the substrate-adsorbate system as the temperature is slowly decreased. The adsorption of methylene blue dye on the target mineral's surface was studied using the Adsorption locator tool, COMPASSIII forcefield parameters, and Monte Carlo simulated annealing. The obtained results go well compared to the previous work^70–73^. The obtained results are displayed in supplementary data (Supplementary Table S2) and Fig. 13.

The adsorption data obtained from the Adsorption Locator could be summarized in the following points:

1. The total energy, adsorption energy, and binding energy reveal the strong affinity of the target mineral toward the working dyes.
2. The value of energy could indicate the nature of the formed bond between the target mineral and the adsorbed dye. The obtained results go well with the Langmuir model, where R_L_ was found in the range of 0.0339–0.584, which indicates a favourable adsorption process.
3. The binding energy was evaluated from the value of the total energy and the optimized energy of the dye and mineral.
4. The working dye was located on the surface of the target substrate as a stacking planar structure, which goes well with the Langmuir model, which gave the most suitable adsorption isotherm model for the adsorption of the MB on the target clay where R^2^ = 0.9971.
5. The interaction of the adsorbed dye with the surface of the target substrate proceeds through the hydrogens and the surface of the aromatic ring.
6. The interaction steps strongly indicate the substrate surface's negative nature and the dye molecule's positive nature.
7. The overall positive nature of the dye molecule pronounced the resonated nature of the dye molecule, which is represented in supplementary data Scheme S1.
8. Cl^-^ was located far away from the surface of the target substrate, giving evidence of the negative nature of the target mineral.
9. The ellipsoid view of the adsorbed dye showed the hydrogens as arms oriented towards the surface of the substrate, also the aromatic ring as stacking positive moiety located and strongly bonded with the surface of the target mineral.
10. The overall fields of the absorbed dye on the surface of the BMNC are presented in Fig. 13.
11. The qualitative view and the estimated energy value go well with the Langmuir model with speculation of the chemisorbed process with some cation exchange behavior.

**Fig. 14:**
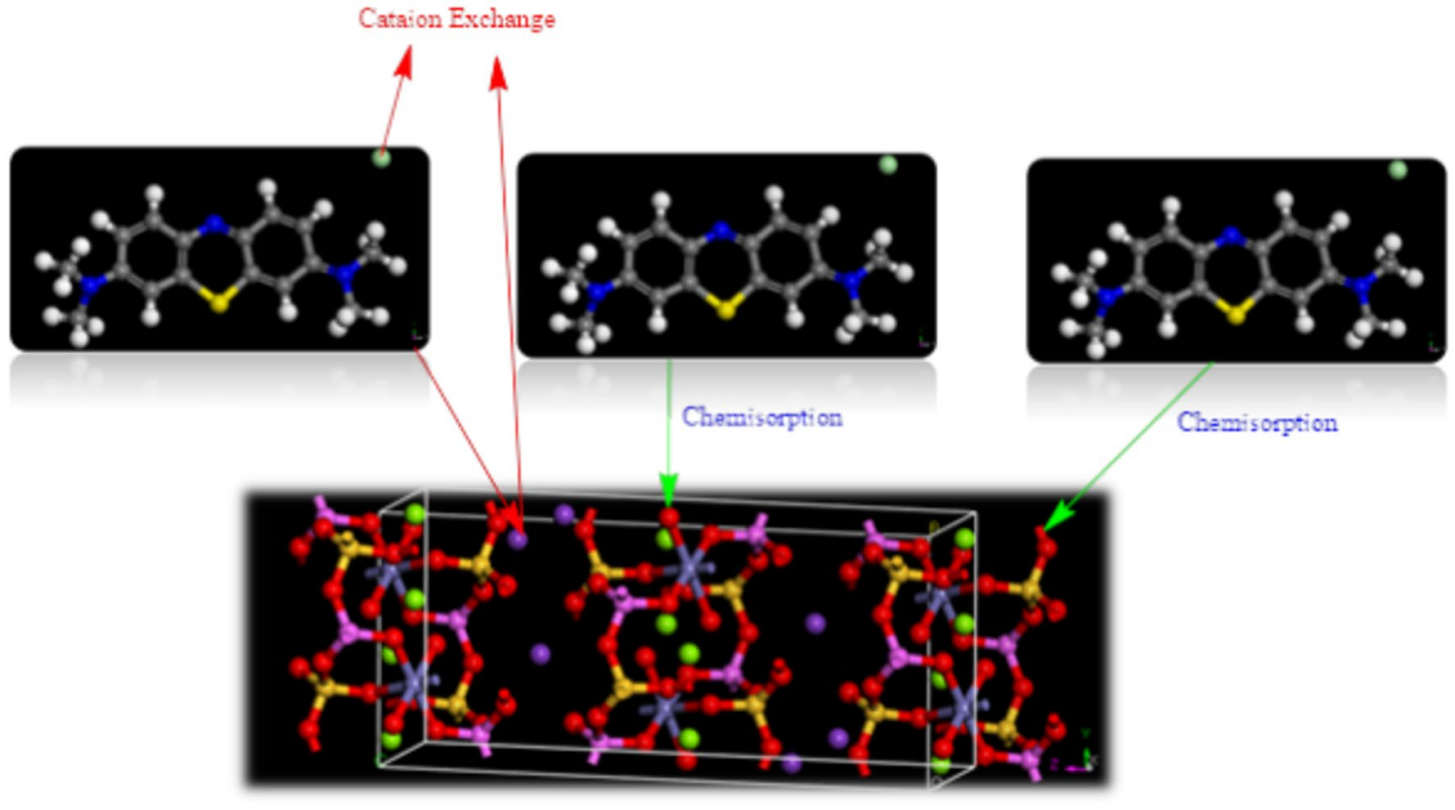
The postulated mechanism of the MB adsorption on BMNC.

According to the experimental and simulated data could be laid out the mechanism of the adsorption of the MB on BMNC as the following Fig. 14.

### Mechanism of adsorption

The mechanism of adsorption of methylene blue (MB) onto ball-milled nano clay (BMNC) was investigated using a combination of analytical techniques and modeling approaches, providing a comprehensive understanding of the process. The following methods were employed:

XRD analysis was conducted to study the crystallinity and phase identification of BMNC. The XRD patterns before and after MB adsorption indicated changes in the crystalline structure of the clay, suggesting interaction between MB molecules and the clay surface. These structural modifications provide evidence of successful adsorption. SEM was used to observe the surface morphology and texture of BMNC before and after adsorption. The SEM images revealed significant morphological changes post-adsorption, indicating the presence of MB on the clay surface. EDS analysis further confirmed the adsorption by detecting the elemental composition, particularly the presence of sulfur from MB on the BMNC surface. The reduction in sodium and potassium elements after adsorption suggests an ion-exchange mechanism. TEM provided high-resolution images of BMNC, showing detailed morphology and particle size at the nanoscale. The TEM images confirmed that the BMNC particles possess a highly fractured and irregular structure, which contributes to the high adsorption capacity due to the increased surface area. This supports the SEM findings and provides a detailed visualization of the adsorbent. The adsorption kinetics were analyzed using pseudo-first-order, pseudo-second-order, and intra-particle diffusion models. The pseudo-second-order model best described the adsorption kinetics, suggesting that chemisorption is the dominant mechanism. Isotherm models, including Langmuir, Freundlich, Temkin, and Dubinin-Radushkevich, were used to analyze the equilibrium data. The Langmuir isotherm model provided the best fit, indicating monolayer adsorption on a homogeneous surface. These models helped elucidate the nature of the interactions between MB and BMNC, confirming that the adsorption process involves both physical and chemical interactions. The pHpzc of BMNC was determined to understand the surface charge properties of the adsorbent. The pHpzc value was found to be in the range of pH 7–8, indicating that at this pH range, the surface of BMNC is optimally charged to attract the cationic dye molecules through electrostatic interactions. This analysis provides insight into the influence of pH on the adsorption capacity and mechanism. FTIR analysis before and after the adsorption of MB on BMNC indicates significant changes in the functional groups, confirming the interaction between the adsorbent and the adsorbate. These changes include shifts in the O–H, Si–O, Al–OH, and H–O–H vibrations, suggesting that multiple interactions such as hydrogen bonding, ion exchange, and complex formation are involved in the adsorption process. The detailed shifts in peak positions and changes in intensity provide valuable insights into the nature of these interactions. Through a combination of FTIR, XRD, SEM, EDS, TEM, kinetic and isotherm modeling, and pHpzc analysis, the adsorption mechanism of methylene blue onto ball-milled nano clay was comprehensively investigated. The structural and morphological analyses confirmed the presence and interaction of MB with BMNC, while the kinetic and isotherm models provided a deeper understanding of the adsorption process. Despite the absence of CHN and XPS analyses, the employed methods were sufficient to elucidate the adsorption mechanism effectively, aligning with findings from existing literature. These results demonstrate that BMNC is an effective adsorbent for MB removal, focused mainly by ion exchange process, supported by the high surface area and favorable surface charge properties of the nano-engineered clay (Fig. 14). This study contributes to the broader understanding of adsorption mechanisms and supports the potential application of BMNC in water treatment processes.

### Comparative study with other sorbents

The advantage of the present study lies in the successful development and application of low cost, green and facile synthesized of BMNC for the efficient removal of MB dye from aqueous solutions. This material demonstrates a high adsorption capacity and effective removal. The combination of these features makes the BMNC nanocomposite a promising and sustainable material for addressing the environmental challenge of MB dye contamination in water. The study contributes significantly to the field of water purification and environmental remediation compared with a number of various adsorbents as shown in Table 5.

**Table 5:** Comparison of adsorption capacities for BMNC and other adsorbents for MB removal.

Adsorbent	Q_max_(mg/g) (dye)	References
Bentonite clay	30.54	^26^
Dolomite clay	202.13	^74^
Clay oued Daraa Valley	70	^46^
Moroccan natural clay	32.5	^44^
Kaolinite clay	102.04	^75^
Modified palygorskite	33.1	^76^
Chefchaouen clay	71.2	^77^
BMNC	128	In this study

## Conclusion

This study demonstrates the efficacy of nano-engineered glauconite clay (BMNC) in the adsorption of methylene blue (MB) from aqueous solutions, highlighting its potential as a practical and sustainable solution for wastewater treatment. The innovative ball-milling process significantly enhances the adsorption properties of glauconite clay, offering a high surface area and numerous active sites for dye removal. The comprehensive analysis using XRD, SEM, EDS, TEM, kinetic and isotherm models, and pHpzc provides a thorough understanding of the adsorption mechanism, confirming both chemisorption and physisorption processes. The novelty of this research lies in the application of a simple yet effective modification technique to a naturally abundant material, transforming it into a powerful adsorbent. The practical applicability of BMNC is evident from its high adsorption capacity, cost-effectiveness, and environmental friendliness. This study not only advances the field of adsorption science but also offers a scalable and sustainable method for addressing the critical issue of dye pollution in industrial wastewater. Future work should focus on the long-term stability and regeneration of BMNC, as well as its effectiveness in removing other contaminants, to further validate its potential for broader environmental applications.

## Supplementary Information

Supplementary Information.

## Data Availability

All data generated through this study are included in this manuscript and the Supplementary data file.
