# Supplementary material for: Advanced nano modification of ecofriendly glauconite clay for high efficiency methylene blue dye adsorption
Source: Sci Rep. 2024 Oct 9;14:23614. doi: 10.1038/s41598-024-71979-y (PMC11464627; doi:10.1038/s41598-024-71979-y)
Supplement: Supplementary file 1 — Supplementary Information. [file 41598_2024_71979_MOESM1_ESM.docx]

**Advanced Nano Modification of Ecofriendly Glauconite clay for High Efficiency Methylene Blue Dye Adsorption**

**Eman M. Saad^1^*, Manar Wagdy^1^, Adel S. Orabi^2^**

^1^ Chemistry Department, Faculty of Science, Suez University, Suez, Egypt

^2^ Chemistry Department, Faculty of Science, Suez Canal University, Ismailia, Egypt

Email: [emmsaad@yahoo.com](mailto:emmsaad@yahoo.com)

|  | a |
| --- | --- |
|  | b |
|  | c |

Figures S1: Non-linear form of kinetic models for adsorption of MB a) 50 mg/L, (b) 100 mg/L and c) 200 mg/L onto BMNC

Figure S2: Non linear isotherm models (Langmuir, Freundlich. Temkin and D-R)


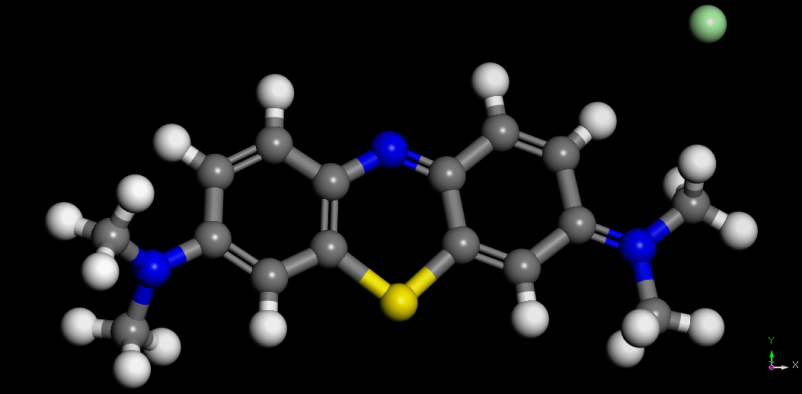


**Figure S3**: Optimized geometry of the methylene blue dye


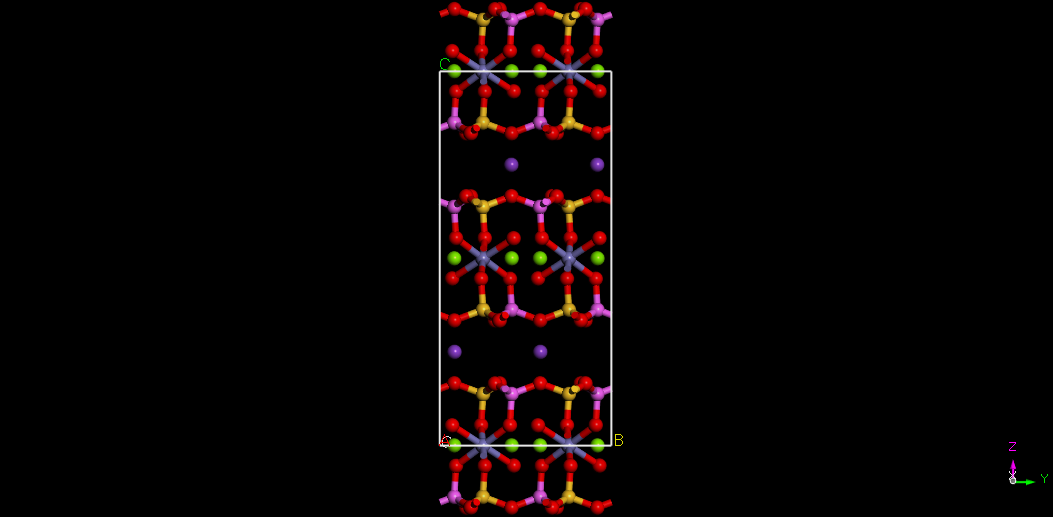

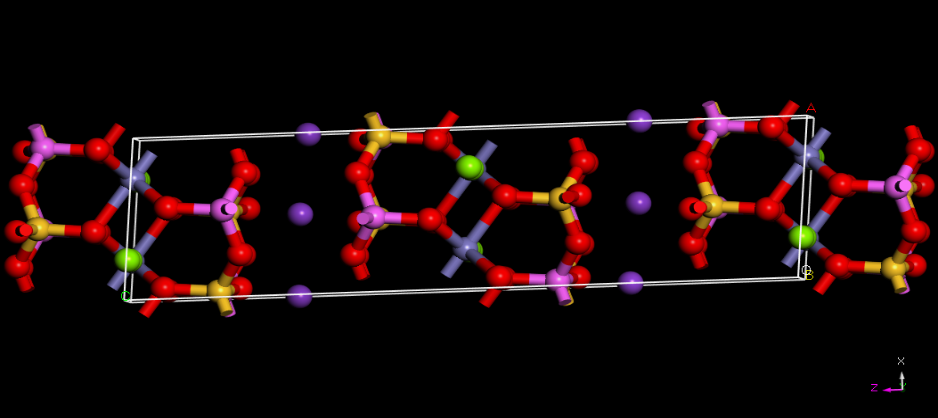

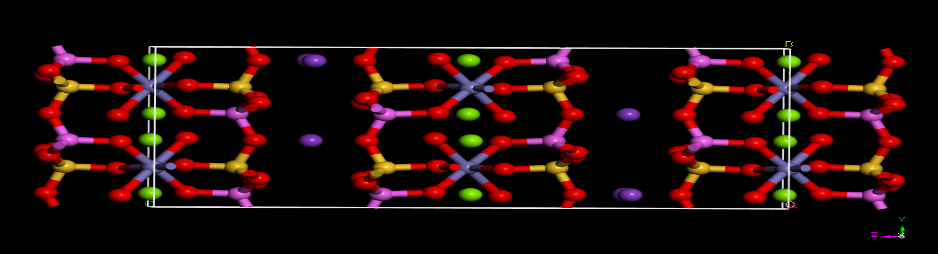


A

C

B

**Figure S4**: (A) The y(→)z(↑), (B) The y(­)z(¬) and (C) The y(→)z(↑) diagrams of the target substrate material: red = O, pink = Al, gray = Fe, yellow = Si, green = Mg or Ca, violet = K or Na

**Scheme S1**: The stepwise resonated structure of the methylene dye

**Table S1 Simulation approach using the EXPO program.**

| Parameter | value | Parameter | value |
| --- | --- | --- | --- |
| a  b  c  α  β  γ | 14.195  13.317  10.262  90.000  90.665  90.000 | Laue group no.  Space Group Number:  Table Setting Choice  Crystal System  Hall Symbol  Hermann-Mauguin Symbol  Laue Group Symbol  Point Group Symbol  Patterson Space Group  Extinction Symbol | 1 P 1 2/m 1  10  b  Monoclinic  -P 2y  P 1 2/m 1  2/m  2/m  P2/m  P 1 - 1 |
| rho min  rho max  d resolution  reflection numbers  h k l (max) | 0.003  0.247  1.006  2112  14 13 10 | List of all symmetry operators: | (1) x, y, z (2) -x, y, -z  (3) -x, -y, -z  (4) x, -y, z |
| Volume | 1939.8 | Z | 2 |

**Table S2 The obtained data of the simulated adsorption process**

| Case  number | Total energy  (kcal/mol) | Adsorption energy  (kcal/mol) | Binding energy  (kcal/mol) |
| --- | --- | --- | --- |
| 1 | -18.735 | -38.426 | -183.785 |
| 2 | -18.283 | -37.975 | -183.333 |
| 3 | -18.082 | -37.774 | -183.132 |
| 4 | -17.807 | -37499 | -182.857 |
| 5 | -17.203 | -36.894 | -182.253 |
| 6 | -17.166 | -36.858 | -182.216 |
| 7 | -16.964 | -36.656 | -182.014 |
| 8 | -16.701 | -36.393 | -181.751 |
| 9 | -16.100 | -35.792 | -181.15 |
| 10 | -15.346 | -35.037 | -180.396 |
